# Sagittal and vertical effects of transverse sagittal maxillary expander (TSME) in three different malocclusion groups

**DOI:** 10.1186/s40510-015-0075-z

**Published:** 2015-04-25

**Authors:** Cinzia Maspero, Guido Galbiati, Lucia Giannini, Giampietro Farronato

**Affiliations:** U.O.C. Chirurgia Maxillo-facciale ed Odontostomatologia (dir: AB. Giannì) Fondazione Cà Granda IRCCS Ospedale Maggiore Policlinico, University of Milan, 20122 Milan, Italy; Maxillo-Facial and Odontostomatology Unit Fondazione Cà Granda IRCCS Ospedale Maggiore Policlinico, University of Milan, 20122 Milan, Italy; UOC Chirurgia Maxillofacciale e Odontostomatologia, Fondazione IRCCS Cà Granda - Ospedale Maggiore Policlinico, University of Milan, Commenda 10, Milano, 20122 Italy

**Keywords:** Rapid maxillary expansion, Maxillary hypoplasia, Hyrax expander, Transverse sagittal maxillary expander

## Abstract

**Background:**

The aim of this retrospective study was to cephalometrically evaluate the skeletal and dental effects of the transverse sagittal maxillary expander (TSME), for the correction of sagittal and transverse maxillary deficiency in class I, II, and III malocclusions.

**Methods:**

The sample for this retrospective study included 45 patients (mean age, 8.4 years; 26 females, 19 males; 15 skeletal class I subjects, 15 skeletal class II subjects, and 15 skeletal class III subjects) with maxillary bilateral cross-bite.

For each patient, a lateral cephalogram was obtained before treatment and at the end of the retention period. Changes in the groups during the observation period were calculated, compared, and statistically analyzed with a *t*-test.

**Results:**

The cephalometric values before T0 and T1 showed significant changes.

**Conclusions:**

The TSME can produce skeletal changes due to the transverse force and sagittal effects on the maxillary alveolar process. These modifications have benefic effects in classes I, II, and III. The data obtained in this study permit us to underline the fact that TSME can be used in all of the skeletal classes, with good vertical and sagittal results.

## Background

Rapid maxillary expansion (RME) is widely used when transverse maxillary deficiency is diagnosed [1-3]. RME not only separates the midpalatal suture but also affects the circumzygomatic and circummaxillary sutural system [3,4]. It has been reported that opening of the midpalatal suture has vertical and sagittal effects on both of the jaws [3]. In 1970, Haas showed marked alteration in growth direction and facial morphology as a result of orthopedic therapy [5]. Many authors found that [5-8] the maxilla is frequently displaced downward and forward during maxillary expansion, while others found only an anterior movement after RME [9,10].

Da Silva et al. confirmed that the maxilla did not show any statistically significant modifications in the sagittal position over the period of activation of the appliance, while it displayed a tendency to rotate downward and backward, increasing the SN-PP angle value [1]. However, Wertz and Dreskin found no significant change in the angulations of the palate with RME therapy [8]. Cleall found unfavorable effects in patients with a well-positioned maxilla reporting that in the retention period it generally returns to its original position [11]. McNamara, in 1993, stated that widening the maxilla led to a spontaneous forward posturing of the mandible during the retention period, correcting the mild class II relationship. He held that it is important to consider the transverse plane prior to the diagnosis of a class II malocclusion [12,13]. The relationship between the transverse dimension and the correction of class II malocclusion was described in 1971 by Reichenbach and Taatz that prove the relationship between the improvement in transverse palatal diameter and the correction of sagittal intermaxillary relationships [14]. In 1889, Kingsley underlined this phenomenon, pointing out how the transverse expansion could favor mandible advancement [15]. This example allows to understand how palatal transverse expansion solves spontaneous mandible repositioning in a forward position, solving or improving sagittal malocclusion. Some authors underlined that in comparing skeletal class I patients with skeletal class II division II patients who have not been orthodontically treated, the second group presents a maxillary and mandible transverse diameter reduction [16].

Many authors observed that maxillary expansion has different effects in class I, II, and III malocclusions [3,17-22]. In the retrospective studies of Farronato et al. on 15 growing subjects with maxillary hypoplasia, the effects of RME in the three planes of space were investigated. The cephalometric tracings were analyzed before and after treatment and at the end of the retention period. The results of their study confirmed widening of the maxilla in the transverse plane and an increase in the floor of the nose. In the sagittal plane, different effects were observed in class I, II, and III subjects [23,24]. In class I patients, ANB angle was slightly affected, while in all class II subjects, it decreased due to forwarding positioning of the mandible, confirming that orthopedic force to the maxillary complex during the early phase of growth can contribute to the correction of class II malocclusions.

The aim of this retrospective study was to cephalometrically evaluate the skeletal and dental effects of the transverse sagittal maxillary expander (TSME), for the correction of sagittal and transverse maxillary deficiency in class I, II, and III malocclusions [25].

## Methods

The sample for this retrospective study included 45 patients (mean age, 8.4 years; 26 females, 19 males; 15 skeletal class I subjects, 15 skeletal class II subjects, and 15 skeletal class III subjects) with maxillary bilateral cross-bite in mixed or permanent dentition.

The inclusion criteria were as follows:

- Caucasian ethnicity;
- No history of orthodontic treatment;
- Growing patients;
- Pretreatment and posttreatment lateral X-ray with excellent contrast;
- Transverse maxillary deficiency (at least 8 mm);
- Sagittal maxillary hypoplasia
- Presence of bilateral posterior cross-bite.

Exclusion criteria were as follows:

- Congenital anomalies;
- Previous orthodontic treatment;
- Dental anomalies

Lateral cephalograms were taken by the same technician [C.R.] with the same machine and manually traced by one operator [L.G.] and verified for landmark location and anatomic contours by a second operator [C.M.]. Any disagreements were solved by retracing the landmark or structure to the mutual satisfaction of both operators.

To exclude intra-operator error, each measurement was repeated by the same operator after a period of 7 days.

The method error was determined using Dahlberg’s formula ME = √∑*d*^2^/2*n*, where *n* is the number of subjects and *d* is the difference between the two measures. The method error did not exceed 0.1 mm for the linear measurements and 0.2° for angular measurements.

Lateral cephalograms were traced by using acetate papers. A lateral cephalogram was taken before treatment (T0) and a second one was taken after retention (T1).

The assessment of the skeletal relationship was based on SNA-SNB, ANB SN^SNP.SNA, SN^GO.GN, S^GO, SNP.SNA^GO.GN, I^SN, and I^FH angles. N-Me and SNP-A were also analyzed.

Class I patients were considered if their ANB angles were between 0° and 4°, class II patients were considered if their ANB angles were greater than 4°, and class III patients were considered if their ANB angles were less than 0°.

**Figure 1:**
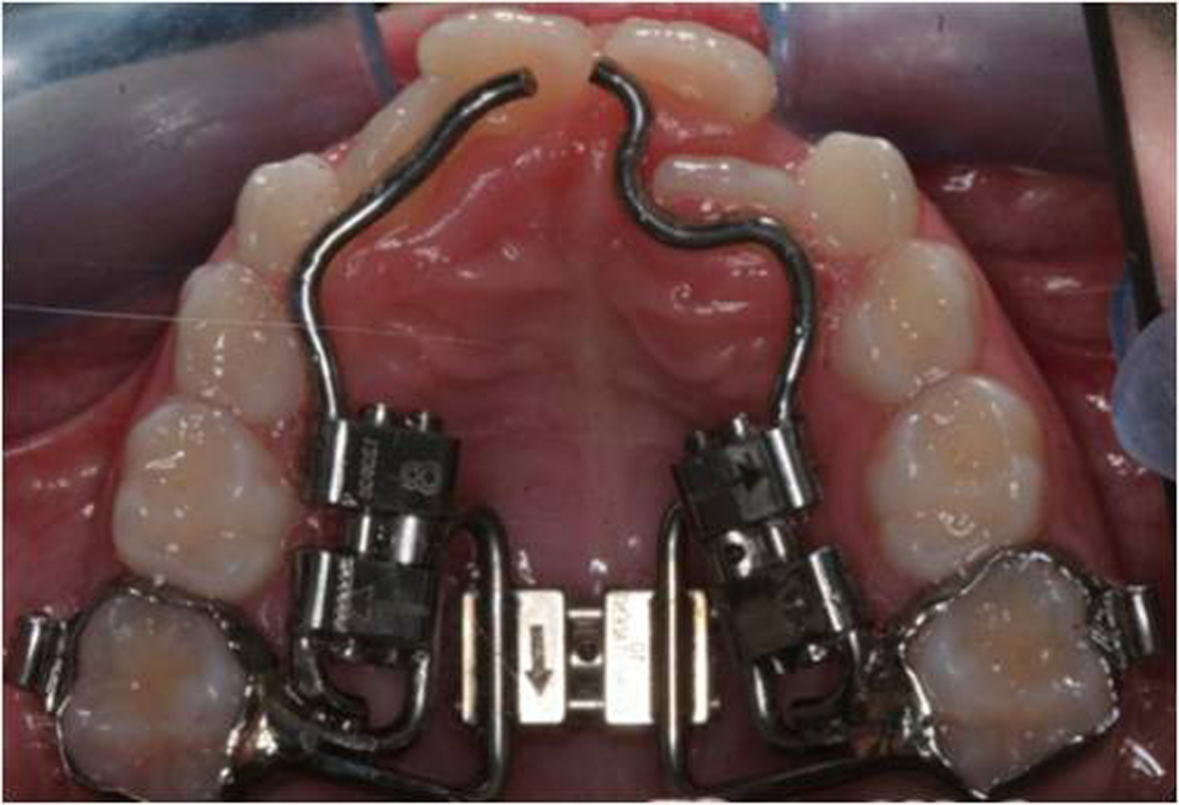
**TSME appliance.**

All the patients were treated by a TSME (Figure 1), a modification of the Hyrax RME, to correct the transverse and sagittal dimensions [25].

The TSME is a fixed device designed to develop arch form in patients with constricted dental arches. It is specifically designed for transverse and anteroposterior arch development. The TSME consists of two bands cemented to the right and left I maxillary molars, a Hyrax-type transverse expansion screw, two .045′′ stainless steel wires extending to the palatal surfaces of the central incisors, and two 8-mm Hyrax-type screws attached to these wires between the molar bands and the incisors. The appliance may also be worn in association with extraoral devices.

The appliance was worn from 8 to 12 months. The protocol of activation consisted in the first phase of activation of the transverse screw one quarter turn twice a day until the desired amount of transverse diameter was obtained. In the second phase, the sagittal screws were activated one quarter turn every 15 days for 6 to 8 months. Then, the appliance was left in place for 4 months of passive retention.

Changes in the cephalometric values during the observation period were calculated and compared.

Descriptive statistics included means and standard deviations (SD). The mean differences in cephalometric measurements before treatment (T0) and after retention period (T1) were examined.

No other treatment took place during the period from T0 to T1 which was the entire first phase treatment.

Statistical analysis has been done using *t*-test for paired samples after checking the normality of the distribution of the data and equality of variances. Statistical significance has been considered for *p* < 0.05.

## Results

Measurements from T0 and T1 in lateral cephalograms for each group are shown in Tables 1, 2, and 3. The cephalometric values before T0 and T1 showed significant changes.

**Table 1:** **Changes in class I patients**

	**Pre**										**Post**									
	**SNA (°)**	**SNB (°)**	**ANB (°)**	**SN-SNPSNA (°)**	**SN-GOGN (°)**	**N.Me**	**S.GO**	**SNP.A**	**I.SN (°)**	**I.FH (°)**	**SNA**	**SNB**	**ANB**	**SN-SNPSNA**	**SN-GOGN**	**N.Me**	**S.GO**	**SNP.A**	**I.SN (°)**	**I.FH (°)**
Average	80.16	78.06	2.1	9.73	33	111.01	64.63	46.34	99.87	112.04	80.26	78.7	1.73	10.43	33.36	111.35	65.02	48.6	102.41	115.01
st.dev.	1.41	1.54	0.89	1.7	4.42	4.28	4.69	4.14	4.32	2.28	1.79	1.75	1.22	1.88	4.23	2.87	4.25	2.05	115.01	2.03
st.anal.	S	S	NS	S	NS	NS	NS	S	S	S										

st.dev., standard deviation; st.anal., statistical analysis; S, significant; NS, not significant.

**Table 2:** **Changes in class II patients**

	**Pre**										**Post**									
	**SNA (°)**	**SNB (°)**	**ANB (°)**	**SN-SNPSNA (°)**	**SN-GOGN (°)**	**N.Me**	**S.GO**	**SNP.A**	**I.SN (°)**	**I.FH (°)**	**SNA**	**SNB**	**ANB**	**SN-SNPSNA**	**SN-GOGN**	**N.Me**	**S.GO**	**SNP.A**	**I.SN (°)**	**I.FH (°)**
Average	79.6	73.93	5.66	9.91	31.23	111.91	65.02	45.56	100.03	110.4	79.96	75.66	4.3	10.93	32.01	110.62	65.55	48.6	103.23	113.51
st.dev.	1.84	1.9	0.77	2.08	2.55	3.67	1.4	3.11	4.82	4.02	1.56	1.27	1.44	1.85	2.54	3.56	1.49	2.98	2.67	3.2
st.anal.	NS	S	S	S	NS	NS	S	S	S	S										

st.dev., standard deviation; st.anal., statistical analysis; S, significant; NS, not significant.

**Table 3:** **Changes in class III patients**

	**Pre**										**Post**									
	**SNA (°)**	**SNB (°)**	**ANB (°)**	**SN-SNPSNA (°)**	**SN-GOGN (°)**	**N.Me**	**S.GO**	**SNP.A**	**I.SN (°)**	**I.FH (°)**	**SNA**	**SNB**	**ANB**	**SN-SNPSNA**	**SN-GOGN**	**N.Me**	**S.GO**	**SNP.A**	**I.SN (°)**	**I.FH (°)**
Average	78.33	81.36	−3.03	9.4	34.26	111.48	64.31	43.26	100.07	109.4	79.2	79.86	−0.66	10.46	34.53	111.71	64.66	47.21	103.07	113.3
st.dev.	2.15	1.83	1.32	2.33	2.32	1.96	2.14	1.83	3.87	2.37	2.23	2.09	1.47	2.22	4.37	1.54	2.21	3.08	2.52	2
st.anal.	S	S	S	S	NS	NS	NS	S	S	S										

st.dev., standard deviation; st.anal., statistical analysis; S, significant; NS, not significant.

### Changes in class I patients

The TSME procedures induced statistically significant modifications in the following cephalometric measurements:

- SNA (+0.10)
- SNB (+0.63)
- SN-SNP.SNA (+0.70)
- SNP.A (+2.26)
- I-SN (+2.55)
- I-FH (+2.97)

SNA increased as a result of an anterior movement of the dentoalveolar maxillary process.

The increase in SNB was due to a forward position of the mandible.

The increase of SN-SNP.SNA resulted in a downward and backward rotation of the palatal plane. SNP-A showed a statistically significant increase as well as the I^SN and I^FH angles. These results were obtained by the activation of the lateral screws during active growth which contribute to the forward movement of the dentoalveolar process and the labial movement of the incisors. SN-Go.Gn and N-Me did not show relevant modifications.

### Changes in class II patients

In class II patients, the TSME procedures induced statistically significant modifications in the following measurements:

- SNB (+1.73)
- ANB (−1.36)
- SN-SNP.SNA (+1.02)
- S.GO (+0.53)
- SNP.A (+3.04)
- I-SN (+3.20)
- I-FH (+3.11)

The increase in SNB was due to a forward position of the mandible. The ANB angle decreased statistically as a result of the forward position of the mandible, improving the skeletal class, and the backward rotation of the palatal plane. In fact, as described for class I patients, the increase in SN-SNP.SNA resulted in a downward and backward rotation of the palatal plane. No increase in the anterior vertical dimension was noted, but a mild decrease in N-Me was noted (20.24) (Table 2). SNP-A, I^SN, and I^FH angles showed a statistically significant increase indicating that the dentoalveolar maxillary process moved anteriorly, because of the force delivered by the sagittal screws and the labial movement of the incisors during active growth.

### Changes in class III patients

The TSME procedures induced statistically significant alterations in the following cephalometric measurements:

- SNA (+0.87)
- SNB (−1.50)
- ANB (+2.37)
- SN-SNP.SNA (+1.06)
- SNP.A (+3.95)
- I-SN (+3.00)
- I-FH (+3.90)

SNA increased as a result of an anterior movement of the dentoalveolar maxillary process.

SNB decreased as a result of the downward and backward rotation of the mandible. The increase in SNA angle and the decrease in SNB angle contributed to the improvement of ANB, which increased in a statistically significant manner. A downward and backward displacement of the palatal plane (SN-SNP.SNA) was observed. A mild increase in the anterior total facial height, N-Me, was also noted, but not statistically, and this caused the downward and backward rotation of the mandible (Table 3). SNP-A and I^SN and I^FH showed a statistically significant increase due to the forward movement of the dentoalveolar process and the labial movement of the incisors.

### Comparison between the three groups

In all of the three groups, SNP-A, I^SN, and I^FH angles showed a statistically significant increase. These results were obtained by the activation of the lateral screws which contribute to the forward movement of the dentoalveolar process and the labial movement of the incisors. Also, SN-SNP.SNA angle increases in all the groups.

ANB showed a different statistically significant variation among the groups.

In class II patients, ANB decreased, while in class III patients, it increased. No statistically significant modifications were found in class I.

SNA increased in a statistically significant manner in class I and III patients.

SNB increased in a statistically significant manner in class II patients and decreased in class III patients. No statistically significant modifications were found for class I.

No statistically significant differences were found in the anterior (N-Me, SN-Go.Gn, and SNP.SNA-GoGn) vertical dimension in any of the three groups.

The posterior vertical dimension (S-Go) remained stable in classes I and III and increased in class II.

## Discussion

Maxillary expansion is indicated in subjects with maxillary narrowness, and it is generally used to increase arch length. Sagittal arch development is indicated when the arch form is constricted since it helps resolve anterior crowding and proclination of the incisors. Labial movement of the anterior teeth may be combined with transverse development of the buccal segments where indicated [26,27].

The TSME is specifically designed for antero-posterior and transverse development. In this study, sagittal and vertical modifications in class I, II, and III growing patients were found after TSME procedures.

Significant modifications were found in the antero-posterior position of the maxillary alveolar process. This change occurred as a result of opening of the mid-palatal suture, bending and movement of the alveolar process anteriorly, and tipping of the incisors [28,29].

In this study, in class II patients, a statistically significant decrease in the ANB angle was obtained during treatment as a result of a statistically significant increase in the SNB angle. These data indicate that in skeletal class II subjects, the constricted maxillary bone impedes physiological sagittal mandibular growth. When the maxillary bone cannot develop normally in the transverse plane as a result of an anomalous function (tongue position, oral breathing), it enhances its development in the vertical plane, with a consequent backward and downward position of the mandible and insufficient and abnormal growth of the nasal septum, which is often deviated. Palatal expansion increases transverse maxillary diameter and releases the mandible, which gains a correct sagittal position. In addition, also the vestibular movement of the upper incisors permits a greater amount of mandibular advancement [30].

The data permit us to underline that class II malocclusions have a strong transverse component. In fact, the expansion of the maxilla disrupts the occlusion determining a slight forward position of the mandible, improving the sagittal occlusal relationship. McNamara suggested that the teeth themselves act as an endogenous functional appliance, encouraging a change in mandibular posture and subsequently a change in the maxillary-mandibular occlusal relationship [12]. According to the same author, this phenomenon usually happens during the first 6 to 12 months of the post-RME period as a result of the gradual repositioning of the lower jaw. Data obtained in this study confirm this theory. In a recent study, Volk et al. concluded that maxillary expansion does not predictably improve dental class II relationship. However, in the study in which 13 class II patients treated by RME were considered, 7 of them improved the dental class II occlusion as well (if not in a statistically significant manner) [27]. The discrepancy between the results obtained in our study and those of Volk et al. is due to the distinct methodologies used. The study of Volk et al. considered the occlusal relationship, in which cast models were mounted in the articulator in centric occlusion and in maximum intercuspation. In skeletal class III patients, a significant anterior movement of the maxilla was found. In fact, after a rapid palatal expansion, thanks to the activation of the circummaxillary suture, a maxillary movement downward and forward can be observed. This translation allows for correction of the skeletal class III occlusion with maxillary retrusion. A slight mandible clockwise rotation aids in the resolution of the sagittal discrepancy. It is important to underline that the correction of skeletal class III occlusion happens during the active phase of therapy, while the correction of skeletal class II occlusion happens during the retention phase [31,32]. This change agrees with the findings in the studies of Wertz [7], Cleall [11], Linder-Aronson and Lingren [21], Davis and Kronman [33], Hicks [34], and Gardner and Kronman [35].

Gardner and Kronman underlined that opening the spheno-occipital synchondrosis could be responsible for the forward displacement of the maxilla [35]. This change happens in the active phase of treatment. The ANB increased by 2.16°; this could be related to the anterior displacement of the maxilla (SNA increased by a statistically significant measure of +0.81°) and the posterior rotation of the mandible. This is in agreement with the findings of Haas [6] and Wertz [7,8]. However, Haas reported that after treatment the maxilla will partially or completely return to its original position. In this group of patients, a slight, but not statistically significant, increase in the mandibular plane angle (SN-Go.Gn) was found [6]. These data agree with the findings of Wertz, who noted that the increase in the mandibular plane angle could be accompanied by a decrease in the SNB angle. A downward and backward displacement of the apical base (SNB) results in a statistically significant rotation of the palatal plane (SN-SNP.SNA) and a slight but not statistically significant rotation of the mandibular plane (Sn- Go.Gn) [7,8]. The increase in the mandibular plane is responsible for the increase in anterior facial height, N-Me, in this group of patients. In class I patients, a slight but statistically significant decrease in the ANB angle and an increase in the palatal plane were found. The difference between the modification in classes I, II, and III of SNA and SNB angles may be due to the amount of transverse expansion needed that is higher in classes II and III in respect to class I. No other statistically significant modifications of the cephalometric measurements studied were found. In all of the patients in each group, SN-SNP.SNA increased as a result of a downward and backward displacement of the palatal plane. The mandibular plane did not show a statistically significant change after TSME procedures, as did the anterior (N-Me) and posterior vertical dimension (S-Go) in all of the groups [36].

The study which has begun from this work can be widened out comparing the data obtained with a control group and increasing the samples size.

## Conclusions

The TSME can produce skeletal changes due to the transverse force and sagittal effects on the maxillary alveolar process. These modifications have benefic effects in classes I, II, and III. The data obtained in this study permit us to underline the fact that TSME can be used in all of the skeletal classes, with good vertical and sagittal results.
